# Supplementary material for: Magnetic-Property Assessment on Dy–Nd–Fe–B Permanent Magnet by Thermodynamic Calculation and Micromagnetic Simulation
Source: Materials (Basel). 2022 Oct 31;15(21):7648. doi: 10.3390/ma15217648 (PMC9657324; doi:10.3390/ma15217648)
Supplement: Supplementary file 1 [file materials-15-07648-s001.zip › materials-1959047-supplementary.pdf]

Supplementary Information

Magnetic-Property Assessment on Dy–Nd–Fe–B Permanent Magnet by Thermodynamic Calculation and Micromagnetic Simulation

Zhiming Dai [1,2], Kai Li [1,3], Zhenhua Wang [1,*], Wei Liu [1] and Zhidong Zhang [1]

[1] Shenyang National Laboratory for Materials Science, Institute of Metal Research, Chinese Academy of Sciences, Shenyang 110016, China

[2] Jiangsu Key Laboratory of Modern Measurement Technology and Intelligent Systems, Department of Physics, Huaiyin Normal University, Huai'an 223300, China

[3] School of Materials Science and Engineering, University of Science and Technology of China, Hefei 230026, China

* Correspondence: zhwang@imr.ac.cn; Fax: +86-24-23891320

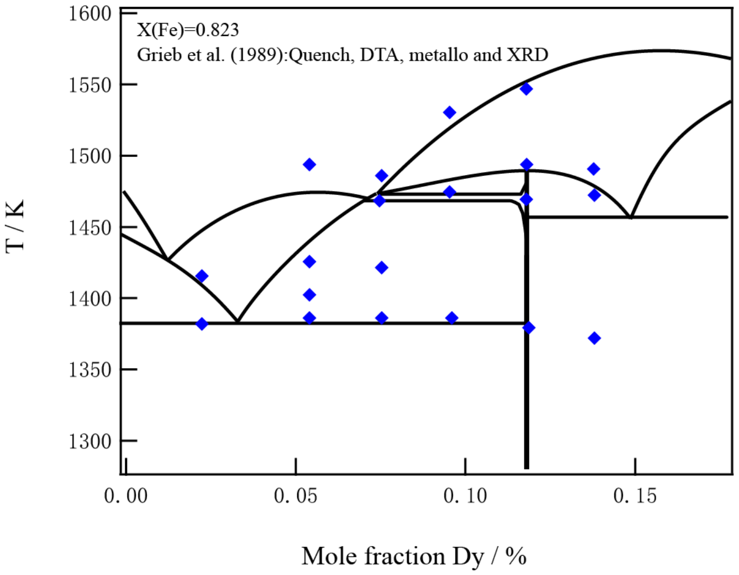

Figure S1. Calculated vertical section for Dy-Fe-B system at 82.3 at% Fe with the experimental data [21].

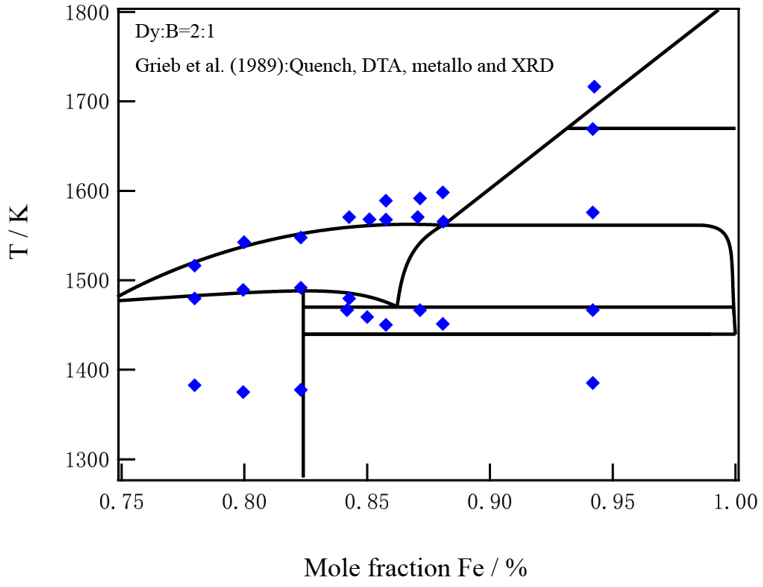

Figure S2. Calculated vertical section for Dy-Fe-B system at Dy:B=2:1 with the experimental data [21].

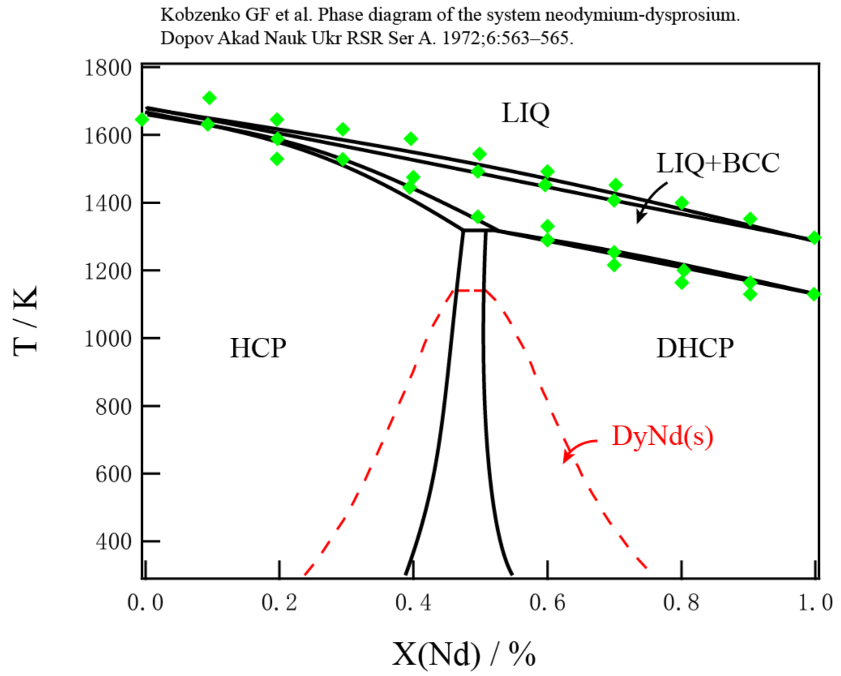

Figure S3. Calculated Nd-Dy binary system with the experimental data [22]. The red dashed line denotes the DyNd stoichiometric compound.

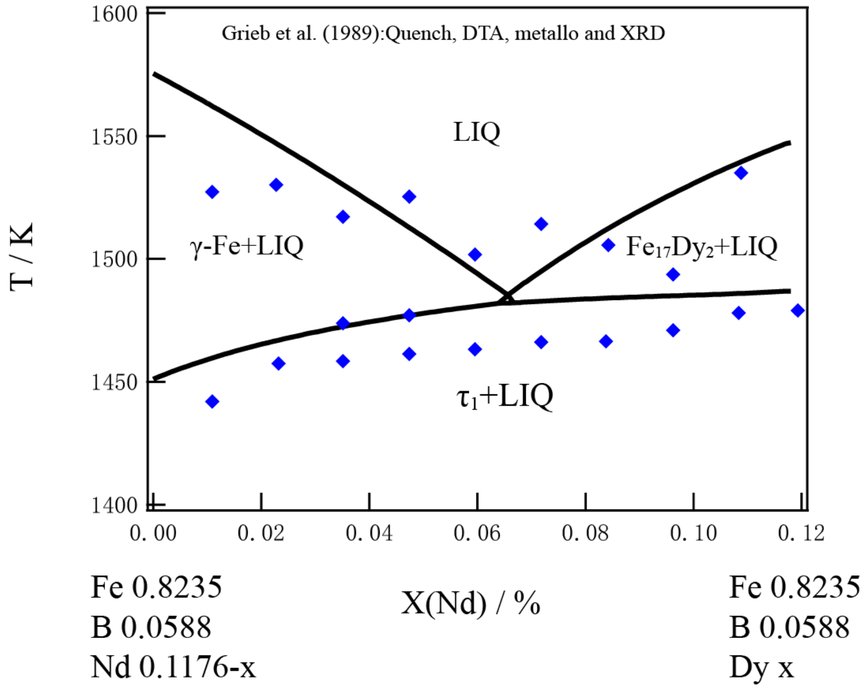

Figure S4. Calculated section of $Nd_2Fe_{14}B$–$Dy_2Fe_{14}B$ with the experimental data [21].

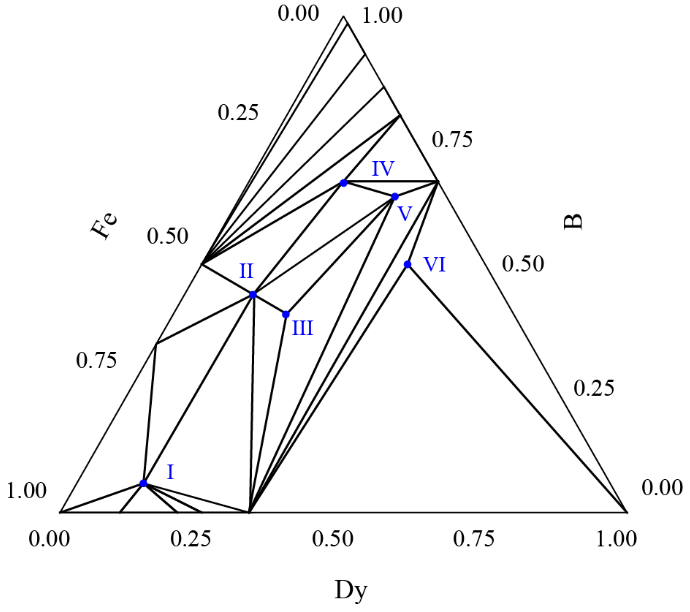

Figure S5. Calculated isothermal section of the Dy-Fe-B ternary system at 1073 K. I-VI denote $Dy_2Fe_{14}B$, $Dy_{1.1}Fe_4B_4$, $DyFe_2B_2$, $DyFeB_4$, Dy_3FeB_7, and Dy_2FeB_3, respectively. The thermodynamic data of $DyFe_2B_2$, $DyFeB_4$, Dy_3FeB_7, and Dy_2FeB_3 stoichiometric phases are not shown in this work for the melting points are absent.

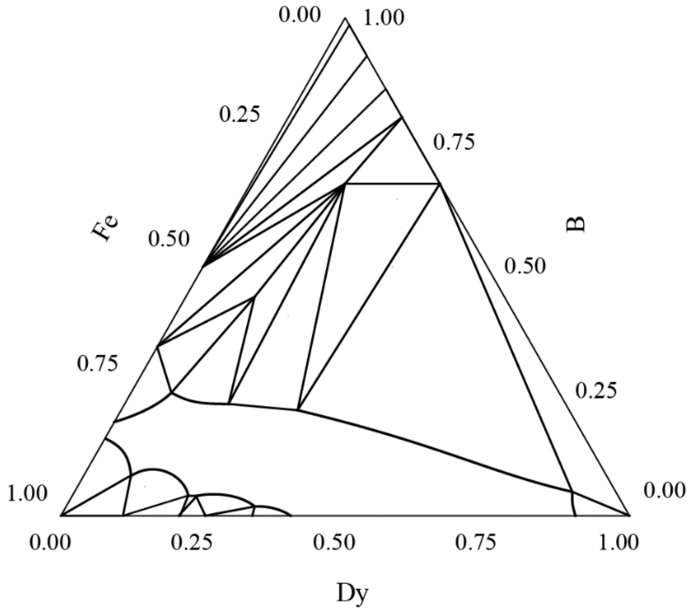

Figure S6. Calculated isothermal section of the Dy-Fe-B ternary system at 1500 K.

References

[21] B. Grieb, G. Schneider, E. T. Henig and G. Petzow, Structural investigations and constitution along $Fe_{14}(Nd_{1-x}(Tb\ or\ Dy)_x)_2B$, Z. Metallkd, 80 (1989) 515–519.

[22] G.F. Kobzenko, V.N. Svechnikov, E.L. Matrynchuk, Phase diagram of the system neodymium-dysprosium. Dopov Akad Nauk Ukr RSR Ser A. 6 (1972) 563–565.
